# Ambulation Distance Within 72 Hours after Surgical Management Is a Predictor of 90-Day Ambulatory Capacity in Elderly Patients with Hip Fracture

**DOI:** 10.5435/JAAOSGlobal-D-23-00079

**Published:** 2023-08-22

**Authors:** Canhnghi N. Ta, Benjamin Lurie, Brendon Mitchell, Roland Howard, Keenan Onodera, Will Harkin, Ryan Ouillette, William T. Kent

**Affiliations:** From the University of California San Diego, Arbor Drive, San Diego, CA (Dr. Ta, Dr. Mitchell, Dr. Howard, Dr. Onodera, and Dr. Kent); the University of Nevada Las Vegas, Las Vegas, NV (Dr. Lurie); the Rush University, Chicago, IL (Dr. Harkin); and the UCLA Health, Santa Monica, CA (Dr. Ouillette).

## Abstract

**Introduction:**

The inability to mobilize after surgical intervention for hip fractures in the elderly is established as a risk factor for greater morbidity and mortality. Previous studies have evaluated the association between the timing and distance of ambulation in the postoperative acute care phase with postoperative complications. The purpose of this study was to evaluate the association between ambulatory distance in the acute postoperative setting and ambulatory capacity at 3 months.

**Methods:**

Patients aged 65 and older who were ambulatory at baseline and underwent surgical intervention for hip fractures from 2014 to 2019 were retrospectively reviewed. Consistent with previous literature, patients were divided into two groups: those who were able to ambulate 5 feet within 72 hours after surgical fixation (early ambulatory) and those who were not (minimally ambulatory).

**Results:**

One hundred seventy patients (84 early ambulatory and 86 minimally ambulatory) were available for analysis. Using a multivariable ordinal logistic regression model, variables found to be statistically significant predictors of ambulatory status at 3 months were the ability to ambulate five feet in 72 hours (*P* < 0.0001), ambulatory distance at discharge (*P* = 0.012), and time from presentation to surgery (*P* = 0.039). Patients who were able to ambulate 5 feet within 72 hours had 9 times the odds of being independent ambulators rather than a lower ambulatory class (cane, walker, and nonambulatory). Pertrochanteric fractures were less likely than femoral neck fractures to independently ambulate at 3 months (17.2% vs. 42.3%; *P* = 0.0006).

**Discussion:**

Ambulating 5 feet within 72 hours after hip fracture surgery is associated with an increased likelihood of independent ambulation at 3 months postoperatively. This simple and clear goal may be used to help enhance postoperative mobility and independence while providing a metric to guide therapy and help counsel patients and families.

Hip fractures are common injuries in the geriatric population and are associated with a decline in mobility and functional independence, as well as notable morbidity and mortality, with 1 year mortality rates after surgery ranging between 15% and 36%.^1,2,3,4,5^ A meta-analysis on hip fracture mortality showed that female and male patients had a 5-fold and 8-fold increase respectively in likelihood of death within the first 3 months after a hip fracture when compared with age-matched and sex-matched controls.^6^ Several studies have found that advanced age, male sex, higher number of preexisting comorbidities, cognitive impairment, and increased time to surgery all negatively impact outcomes in patients with hip fractures.^2,4,7,8,9,10,11,12,13,14,15,16^

Early mobilization and physical therapy in the acute care phase after surgery have been shown to minimize complications, enhance recovery, and help patients to return to independent function after a hip fracture.^17-19^ The ability to ambulate within the first or second postoperative day has been associated with accelerated functional recovery and fewer discharges to high-level care facilities.^20^ Conversely, delays in mobilization have been associated with poor functional outcomes at 2 months after surgery and lower 6-month survival.^21^ However, whether immediate postoperative mobility is associated with improved ambulatory capacity during longer term outpatient follow-up has not been clearly shown, and additional longer term functional outcomes are needed.^22^

Some studies have evaluated the association between the timing and distance of ambulation in the postoperative acute care phase with postoperative complications. In a recent study, it was found that ambulating more than 5 feet within 72 hours postoperatively was associated with decreased complications, morbidity, and mortality.^23^ The authors proposed this threshold as a clinically relevant measure that could be used to optimize postoperative rehabilitation and outcomes. Other studies have attempted to establish a correlation between immediate postoperative function and ambulatory capacity during follow-up; however, these studies evaluated only 1-month postoperative follow-up and lacked specific details in ambulatory capacity, fracture type, and the type of surgical fixation in their analyses.^24,25^ The purpose of this study was to investigate whether the ability to ambulate 5 feet within 72 hours of surgery is associated with independent ambulatory capacity at 3 months in surgically treated patients with hip fracture. Secondary aims included identifying additional predictors of 3-month ambulatory capacity such as age, sex, comorbidities, the type of fracture, surgical technique, implant used, and time to surgery. We hypothesized that using multivariable modeling could predict a patient's 3-month ambulatory status from simple perioperative variables and thereby identify patients at risk for poor functional recovery.

## Methods

After Institutional Review Board approval, a retrospective review using current procedural terminology (CPT) codes was performed to identify all patients at least aged 65 years who were treated surgically for a hip fracture (defined as a fracture of the femoral neck, intertrochanteric, or subtrochanteric region) from February 2014 to May 2019 at a Level I trauma center. Exclusion criteria included patients with less than 80 days of follow-up after surgery (to allow for variation in scheduling of our standard 3-month clinic visit), pathologic fractures, and periprosthetic fractures.

A review of the electronic medical record was performed to obtain demographic data, medical comorbidities, concurrent injuries, mechanism of injury, body mass index, length of hospitalization, and baseline ambulatory status. Ambulatory status was divided into four groups based on standard documentation by the Orthopaedic Trauma service: independently ambulatory without assistive devices, ambulatory with cane, ambulatory with walker, and nonambulatory. Radiographs and CT, if available, were reviewed to determine fracture type. Fractures were classified as fractures of the femoral neck, intertrochanteric region, or subtrochanteric region (fractures within 5 centimeters of the lesser trochanter), with intertrochanteric and subtrochanteric fractures grouped together as pertrochanteric fractures for analysis. Fractures were also further characterized by Garden or arbeitsgemeinschaft für osteosynthesefragen (AO)/orthopaedic trauma association (OTA) classifications. Surgical notes and postoperative imaging were reviewed to determine the type of implant used (closed reduction with percutaneous fixation with three cannulated screws [CRPF], sliding hip screw [SHS], cephalomedullary nail [CMN], or arthroplasty). Immediate postoperative ambulatory capacity was obtained from inpatient physical therapy notes, with data including timing from surgery to ambulation, distance of first ambulation, and maximum ambulatory distance achieved during inpatient hospitalization. Postoperative physiotherapy was initiated within 24 hours of surgical intervention for all patients. Patients were divided into two groups: those who were able to ambulate 5 feet within 72 hours after surgery (early ambulatory) and those who were not (minimally ambulatory). Ambulatory capacity at 3 months was the primary outcome and was obtained through a review of follow-up clinic visit notes.

A statistical analysis was performed using Python. Continuous data were tested for normality using a D'Agostino-Pearson test. A nonparametric Mann-Whitney *U* test was used to compare all continuous variables because of most variables being non-normally distributed. The chi-squared test was used to compare observed versus expected frequencies. The chi-squared test was also used to compare frequencies in categorical variables with more than two levels (such as ambulatory status). To identify independent predictors of ambulatory status at three months, a multivariable ordinal logistic regression model was developed. Ordinal logistic regression was used to allow the prediction of a categorical outcome variable with a known order (ambulatory status at 3 months), rather than collapsing the outcome into two variables as would be done in binary logistic regression or assuming a linear relationship between outcome categories as would be done in linear regression. Owing to the multicollinearity between the variables, ‘fixation type’ and ‘fracture type’ (which occurred because of only femoral neck fractures being treated with arthroplasty at our institution), fracture type was not included in the final multivariable ordinal regression model to avoid the associated problems of unstable coefficient estimates, coefficient sign flipping, and model overfitting.

## Results

One hundred and seventy patients met the inclusion criteria with a median follow-up of 4.3 months (IQR = 3.48 to 5.85 months). The mean age was 80.3 ± 9.1 years and 67.6% of the study patients were women. Before injury, 101 (59%) patients were independent community ambulators, 23 (14%) ambulated with a cane, and 46 (27%) ambulated with a walker. There were 71 femoral neck fractures and 99 pertrochanteric (86 intertrochanteric, 13 subtrochanteric) fractures. 86 patients were treated with a short or long CMN, 29 with three cannulated screws, 20 with a SHS, and 35 patients with arthroplasty (34 hemiarthroplasty, one total hip arthroplasty). All patients were instructed to be weight-bearing as tolerated postoperatively.

Eighty-four patients (49.4%) were able to ambulate at least 5 feet within 72 hours of surgery (the early ambulatory group), whereas 86 patients were unable to do so (the minimally ambulatory group). The groups were similar in sex distribution, Charleston Comorbidity Index, time from injury to surgery, and time from surgery to the final follow-up (*P* > 0.05) (Supplemental Table, http://links.lww.com/JG9/A297). The minimally ambulatory group was significantly older (82.5 vs. 78.0 years; *P* = 0.001) and included more pertrochanteric than femoral neck fractures (65.1% vs. 51.2%; *P* = 0.02). The minimally ambulatory group had a lower proportion of preinjury independent ambulators compared with the early ambulatory group (47.7% vs 71.4%; *P* = 0.006). The early ambulatory group had a slightly longer time from presentation to surgery (1.15 days versus 0.84 days, *P* = 0.242). There was a difference in the type of surgical fixation used in the minimally ambulatory group versus the early ambulatory group: 58.1% versus 42.9% of patients were treated with a CMN, 24.4% versus 16.6% with arthroplasty, 11.6% versus 11.9% with a SHS, and 5.8% versus 28.6% with three cannulated screws (*P* = 0.001 on multiple chi-squared).

A subanalysis separating patients by fracture type (pertrochanteric or femoral neck) was also performed. Regarding preinjury functional status, the femoral neck group had a greater proportion of patients who were independent ambulators before injury than the pertrochanteric group (71.8% vs. 50.5%) and a lower number of patients with using a walker or cane (28.2% vs. 49.5%, *P* = 0.003). In the pertrochanteric group, there was no notable difference in the proportion of patients who received a CMN (rather than a SHS) in the minimally ambulatory versus early ambulatory groups: six patients (10.7%) of minimally ambulatory patients were treated with a SHS and 50 (89.3%) with a CMN, whereas seven patients (16.3%) in the early ambulatory group were treated with a SHS and 36 (83.7%) with a CMN. In the femoral neck group, the minimally ambulatory group had a greater proportion of patients treated with arthroplasty than the early ambulatory group: (70% vs. 34.1% arthroplasty), whereas the early ambulatory group had a greater number of patients who were treated with cannulated screws (58.5% vs. 16.7%). The rates of SHS were more similar between the two groups. In the femoral neck group, 57.7% of patients were able to ambulate 5 feet within 72 hours versus 43.4% of patients in the pertrochanteric group (*P* = 0.07). There was a significant difference in the 3-month ambulatory status between femoral neck and pertrochanteric fractures: 42.3% of femoral neck patients were independent ambulators versus 17.2% of pertrochanteric patients (*P* = 0.0006 on multiple chi-squared) (Table 1).

**Table 1:** Three-Month Ambulatory Status Between Femoral Neck and Pertrochanteric Fractures

	Femoral Neck (n = 71)	Pertrochanteric (n = 99)	*P*
Ambulatory status			0.0006*
Independent	30 (42.3%)	17 (17.2%)	
Cane	2 (2.8%)	10 (10.1%)	
Walker	33 (46.5%)	68 (68.7%)	
Nonambulatory	6 (8.4%)	4 (4%)	

*p< 0.05

At 3 months after surgery, patients in the early ambulatory group had a greater proportion of independent ambulators than the minimally ambulatory group (47.6% versus 8.1%) and a lower proportion of patients with using a walker (36.9% versus 81.4%) (*P* < 0.00001 on multiple chi-squared). This finding persisted at 6 months after surgery (*P* = 0.0002) in the remaining 76 patients (44.7% follow-up). At the longest available follow-up, six patients in each cohort underwent revision surgery (*P* = 0.674). In the early ambulatory group, three of the reoperations were because of osteonecrosis from closed reduction percutaneous pinning (CRPP) of femoral neck fractures, two were from screw cutout from dynamic hip screw (DHS), and one was because of painful hardware from a CMN. In the minimally ambulatory group, three reoperations were attributed to screw cutout, two from painful hardware with CMNs, and one prosthetic joint infection after a hemiarthroplasty (Supplemental Table, http://links.lww.com/JG9/A297).

## Prediction of 3 Months Ambulatory Status

A multivariable ordinal logistic regression model was developed to predict ambulatory status at 3 months, with ambulatory status treated as an ordinal variable with four categories: nonambulatory, ambulatory with a walker, ambulatory with a cane, and ambulatory without assistance (Table 2). The model was provided with 11 potential predictors: sex, age, Charleston Comorbidity Index, preinjury ambulatory status, time from presentation to surgery, time from injury to surgery, surgical treatment with either arthroplasty or internal fixation, ability to ambulate five feet within 72 hours, ambulatory distance at first postoperative evaluation, ambulatory distance at discharge, and need for revision surgery. The only variables found to be statistically significant independent predictors of ambulatory status at 3 months were ability to ambulate five feet in 72 hours (*P* < 0.0001), ambulatory distance at discharge (*P* = 0.012), and time from presentation to surgery (*P* = 0.039). Compared with the minimally ambulatory group, the early ambulatory group had 8.9 times the odds of being independent ambulators rather than a lower ambulatory class (cane, walker, nonambulatory), holding all other variables constant. For every day of delay between patient presentation and surgery, patients had 0.79 times the odds of being an independent ambulator (greater odds of using a cane, walker, or nonambulatory). Finally, every foot that a patient was able to ambulate before discharge was associated with 1.01 times greater odds of being an independent ambulator at 3 months. Higher preinjury ambulatory status trended toward positively predicting 3-month ambulatory status (OR 1.42, *P* = 0.13).

**Table 2:** Multivariable Ordinal Logistic Regression Model for Ambulatory Status at 3 months

Variable	Coefficient	Standard Error (SE)	P > |z|	95% Confidence Interval (CI)
Sex - Female	1.027	0.373	0.943	[−0.704, 0.757]
Arthroplasty or open reduction internal fixation (ORIF)	0.608	0.47	0.291	[−1.417, 0.425]
5 ft. within 72 hours - No	0.112	0.498	0*	[−3.158, −1.204]
Revision surgery - No	1.823	0.687	0.382	[−0.746, 1.947]
Age	0.970	0.022	0.17	[−0.073, 0.013]
CCI	0.966	0.086	0.688	[−0.203, 0.134]
Postoperative ambulatory distance	0.992	0.006	0.21	[−0.02, 0.004]
Ambulatory distance at discharge	1.012	0.005	0.012*	[0.003, 0.023]
Time from injury to surgery	1.012	0.051	0.815	[−0.088, 0.112]
Time from presentation to surgery	0.787	0.116	0.039*	[−0.466, −0.012]
Preinjury functional status	1.417	0.227	0.125	[−0.097, 0.795]

*p< 0.05

To generate an estimate of model accuracy and to assess for overfitting, 1,000 models were trained on a random subset of 75% of patients and their ability to correctly predict ambulatory status at 3 months was then tested on the remaining 25% of unseen patients. The median accuracy of the model at correctly predicting a patient's specific ambulatory status (independent, cane, walker, nonambulatory) using this technique was 70% (compared with the 25% accuracy expected by chance alone).

## Discussion

Immobility after geriatric hip fractures has been consistently identified as a risk factor for future morbidity and mortality. One of the central goals of surgical intervention is to allow early ambulation and mobilization. The goal of this study was to determine whether a patient's ability to ambulate greater than 5 feet within the first 72 hours after surgery (the early ambulatory group) could predict their ambulatory status at 3 months. Secondary aims were to identify other potential predictors of ambulatory status at 3 months and to examine the impact of fracture pattern and type of fixation used.

Geriatric hip fractures have high rates of morbidity and mortality, and patients are frequently unable to return to their preinjury level of function.^26^ Early postoperative mobilization is important for preventing complications and regaining functional independence.^27,28^ Numerous studies have evaluated how patient and surgery specific factors can influence the postoperative course. Furthermore, short-term postoperative mobility has been associated with decreased complications rates, morbidity and mortality.^3,4,18,21,22,23,24,25^ Although postoperative exercise and physical therapy have been shown to improve independent function, to our knowledge no prior studies have evaluated the relationship between both postoperative timing and distance of ambulation with subsequent ambulatory independence to help delineate optimal intervention programs.

To help provide clear postoperative goals and expectations, VanTienderen et al^23^ performed a retrospective study evaluating the effect of ambulatory distance and timing on morbidity and mortality in the immediate postoperative period. The authors found that ambulating 5 feet within 72 hours after hip fracture fixation was associated with a decreased risk of 90-day morbidity and mortality.^23^ Patients that were able to walk more than 5 feet within 72 hours postoperatively had a complication rate (ie, pneumonia, intensive care unit (ICU) admission, deep vein thrombosis (DVT), death) of 31% versus 77% in those that were unable to.

In the present study, we sought to examine the relationship between immediate postoperative ambulatory capacity and ambulatory status 3 months after surgery. Using multivariable ordinal regression, three independent predictors of ambulatory status at 3 months were identified: ability to ambulate 5 feet within 72 hours after surgery, greater ambulatory distance with physical therapy at discharge, and shorter time from presentation to surgery. The early ambulatory group had nearly 9 times the odds of being an independent ambulator rather than a lower ambulatory ability compared with the minimally ambulatory group. Similarly, for each additional foot ambulated with physical therapy on the day of discharge, patients had 1.01 times the odds of being an independent ambulator, which is equivalent to a patient who ambulates 50 feet having approximately 1.64 times greater odds of being an independent ambulator than a patient who ambulates one foot. Regarding timing of surgery, each additional day from presentation to surgery was associated with a 0.79 times lower odds of independent ambulation at 3 months, reaffirming the importance of treating hip fractures surgically within 48 hours when possible to minimize complications and improve outcomes.^15,29,30^ While multivariable modeling aims to derive the independent impact of each variable, time from presentation to surgery may still be confounded by patients with worse preoperative functional status and more comorbidities requiring additional pre-operative optimization.^31^

To assess the accuracy of our model, the train and test technique was used. We iteratively trained 1,000 models on a portion of the data, and then examined the ability of the models to predict specific ambulatory capacity at 3 months. The median accuracy of the models at correctly predicting ambulatory status (specifically predicting whether a patient would use no device, a cane, a walker, or be non-ambulatory) was 70%, demonstrating that the models were able to predict accurately even on data they had never seen before. While our 6-month follow-up was limited by small sample size, the remaining patients in the early ambulatory continued to have a greater proportion of independent ambulators than the minimally ambulatory group, suggesting our results would likely follow this trend at longer term follow up. Collectively, the findings of this study provide several perioperative variables that can reliably predict a patient's future ambulatory function. This knowledge can be used to identify patients at risk for poor post-operative mobility and allows for allocation of appropriate support to optimize postoperative outcomes in hip fracture patients. It may further be used to set expectations with patients and their families when providing perioperative counseling.

In addition to multivariable modeling, we directly compared other clinically relevant perioperative variables between the early ambulatory and minimally ambulatory groups. The minimally ambulatory group had a greater proportion of pertrochanteric fractures, while the early ambulatory group composed of more femoral neck fractures. This supports the existing literature which has found that patients with femoral neck fractures have higher overall locomotion, transfer, and self-care functional independence measure scores at 2 months postoperatively when compared with pertrochanteric fractures.^32,33^ This is consistent with our finding that patients with femoral neck fractures had a greater proportion of independent ambulators at 3 months. This is likely at least partly due to these injuries occurring in different patient populations, with prior studies demonstrating a greater incidence of pertrochanteric fractures in older patients with lower baseline function status.^34-36^ However, fracture location with resultant indicated surgery and pain control are the suspected primary contributing factors to delayed mobility with pertrochanteric fractures. Stable femoral neck injuries, including incomplete, non-displaced or valgus impacted fractures that undergo CRPF, typically have better outcomes and femoral neck fractures that require arthroplasty often result in significantly less pain.^37-39^ It is likely that these patients have fewer barriers to early mobilization, which is consistent with our finding that significantly more femoral neck fracture patients, who largely underwent CRPF or arthroplasty, were independently ambulatory at 3 months compared with the pertrochanteric group. Lastly, while the early ambulatory group had a higher proportion of patients treated with CRPF and lower proportion treated with arthroplasty, the fracture patterns are not uniform between the groups, and this is not suggestive of CRPF being a superior treatment method. Prior literature has demonstrated high revision rates after internal fixation in elderly patients with femoral neck fractures, particularly those with displacement.^40^

## Limitations

As a retrospective chart review, this study has important limitations to consider. Regarding preinjury ambulatory status and comorbidities, we were reliant on available documentation in the electronic medical records (EMR) and were not able to prospectively collect the data. There was also no prospective standardization of surgical technique or implant choice between the treating traumatologists at our institution. This study did not include an assessment of reduction quality, postoperative pain, or postoperative mental status, all of which could affect ambulatory status at the 3-month follow-up and would be targeted with very different interventions to improve outcomes. We were also not able to determine whether there were variations in the physical therapy that patients received about technique, time spent, or therapist resources. Because femoral neck fractures and pertrochanteric fractures are treated with different implants and surgical techniques (namely, arthroplasty being exclusively used for the treatment of femoral neck fractures), differentiating the impact of fracture type versus implant type on functional outcomes was difficult. It is also possible that the impact of fracture pattern and implant used might change over longer follow-up because patients continue to recover, experience failures of fixation, or develop nonunions. Similarly, the short-term follow-up of the study could be addressed by future prospective studies that include outreach to this group of patients that often have difficulty maintaining normal scheduled follow-up. Last, as stated by VanTienderen et al,^23^ it is possible that a more optimal combination of postoperative time to ambulation and ambulation distance exists to better predict short-term and long-term outcomes. However, we believe this straightforward perioperative metric provides important information to patients and to clinicians working to optimize patient outcomes after hip fracture surgery.

## Conclusion

This study demonstrated that patients with hip fracture who can ambulate 5 feet within 72 hours of surgery have nearly 9 times the odds of being an independent ambulator at 3 months compared with those who cannot ambulate in the immediate postoperative period. In addition to describing 72-hour postoperative ambulatory status as a novel predictor of ambulatory status at 3 months, we confirmed the findings of previous studies: increased time from patient presentation to surgery negatively predicts functional outcome, as does having a pertrochanteric rather than a femoral neck fracture. The findings from this study can be used by clinicians, patients, and their families to better understand a given patient's expected postoperative course and level of independence within just 72 hours of surgical intervention. Future interventional studies aimed at improving patients' immediate postoperative ambulatory status could help determine whether interventions during the immediate postoperative period can improve functional outcomes of hip fracture patients.

## Supplementary Material

## References

[1] Civinini R Paoli T Cianferotti L : Functional outcomes and mortality in geriatric and fragility hip fractures-results of an integrated, multidisciplinary model experienced by the “Florence hip fracture unit”. Int Orthop 2019;43:187-192. 30159804 10.1007/s00264-018-4132-3

[2] Pioli G Lauretani F Davoli ML : Older people with hip fracture and IADL disability require earlier surgery. Journals Gerontol Ser A: Biol Sci Med Sci 2012;67:1272-1277. 10.1093/gerona/gls097 22454376

[3] Tarazona-Santabalbina FJ Belenguer-Varea Á Rovira-Daudi E : Early interdisciplinary hospital intervention for elderly patients with hip fractures: Functional outcome and mortality. Clinics (Sao Paulo) 2012;67:547-555. 22760891 10.6061/clinics/2012(06)02 PMC3370304

[4] Mariconda M Costa GG Cerbasi S : The determinants of mortality and morbidity during the year following fracture of the hip: A prospective study. Bone Joint J 2015;97-B:383-390. 25737523 10.1302/0301-620X.97B3.34504

[5] Lee TC Ho PS Lin HT Ho ML Huang HT Chang JK: One-year readmission risk and mortality after hip fracture surgery: A national population-based study in taiwan. Aging Dis 2017;8:402-409. 28840055 10.14336/AD.2016.1228 PMC5524803

[6] Haentjens P Magaziner J Colón-Emeric CS : Meta-analysis: Excess mortality after hip fracture among older women and men. Ann Intern Med 2010;152:380-390. 20231569 10.1059/0003-4819-152-6-201003160-00008 PMC3010729

[7] Kim SM Moon YW Lim SJ : Prediction of survival, second fracture, and functional recovery following the first hip fracture surgery in elderly patients. Bone 2012;50:1343-1350. 22421579 10.1016/j.bone.2012.02.633

[8] Smith T Pelpola K Ball M Ong A Myint PK: Pre-operative indicators for mortality following hip fracture surgery: A systematic review and meta-analysis. Age Ageing 2014;43:464-471. 24895018 10.1093/ageing/afu065

[9] Brown K Cameron ID Keay L Coxon K Ivers R: Functioning and health-related quality of life following injury in older people: A systematic review. Inj Prev 2017;23:403-411. 28073948 10.1136/injuryprev-2016-042192

[10] Haentjens P Magaziner J Colon-Emeric C : Meta-analysis: Excess mortality after hip fracture among older women and men. Ann Intern Med 2010;152:380-390. 20231569 10.1059/0003-4819-152-6-201003160-00008 PMC3010729

[11] Chang W Lv H Feng C : Preventable risk factors of mortality after hip fracture surgery: Systematic review and meta-analysis. Int J Surg 2018;52:320-328. 29530826 10.1016/j.ijsu.2018.02.061

[12] Espinosa KA Gélvez AG Torres LP García MF Peña OR: Pre-operative factors associated with increased mortality in elderly patients with a hip fracture: A cohort study in a developing country. Injury 2018;49:1162-1168. 29674111 10.1016/j.injury.2018.04.007

[13] Frost SA Nguyen ND Center JR Eisman JA Nguyen TV: Excess mortality attributable to hip-fracture: A relative survival analysis. Bone 2013;56:23-29. 23684802 10.1016/j.bone.2013.05.006

[14] Rosso F Dettoni F Bonasia DE : Prognostic factors for mortality after hip fracture: Operation within 48 hours is mandatory. Injury 2016;47 suppl 4:S91-S97. 27546722 10.1016/j.injury.2016.07.055

[15] Moja L Piatti A Pecoraro V : Timing matters in hip fracture surgery: Patients operated within 48 hours have better outcomes. A meta-analysis and meta-regression of over 190,000 patients. PLoS One 2012;7:e46175. 23056256 10.1371/journal.pone.0046175 PMC3463569

[16] Borges FK Bhandari M Guerra-Farfan E : Accelerated surgery versus standard care in hip fracture (HIP ATTACK): An international, randomised, controlled trial. Lancet 2020;395:698-708. 32050090 10.1016/S0140-6736(20)30058-1

[17] Penrod JD Boockvar KS Litke A : Physical therapy and mobility 2 and 6 months after hip fracture. J Am Geriatr Soc 2004;52:1114-1120. 15209649 10.1111/j.1532-5415.2004.52309.x PMC1454714

[18] Beaupre LA Binder EF Cameron ID : Maximising functional recovery following hip fracture in frail seniors. Best Pract Res Clin Rheumatol 2013;27:771-788. 24836335 10.1016/j.berh.2014.01.001 PMC4610717

[19] Lee KJ Um SH Kim YH: Postoperative rehabilitation after hip fracture: A literature review. Hip Pelvis 2020;32:125-131. 32953704 10.5371/hp.2020.32.3.125 PMC7476786

[20] Oldmeadow LB Edwards ER Kimmel LA Kipen E Robertson VJ Bailey MJ: No rest for the wounded: Early ambulation after hip surgery accelerates recovery. ANZ J Surg 2006;76:607-611. 16813627 10.1111/j.1445-2197.2006.03786.x

[21] Siu AL Penrod JD Boockvar KS Koval K Strauss E Morrison RS: Early ambulation after hip fracture: Effects on function and mortality. Arch Intern Med 2006;166:766-771. 16606814 10.1001/archinte.166.7.766 PMC3045760

[22] Handoll HH Sherrington C Mak JC: Interventions for improving mobility after hip fracture surgery in adults. Cochrane Database Syst Rev 2011:CD001704. 21412873 10.1002/14651858.CD001704.pub4

[23] VanTienderen RJ Fernandez I Reich MS Nguyen MP: Walking greater than 5 feet after hip fracture surgery is associated with fewer complications, including death. J Am Acad Orthop Surg 2021;29:213-218. 32694327 10.5435/JAAOS-D-19-00320

[24] Kim JL Jung JS Kim SJ: Prediction of ambulatory status after hip fracture surgery in patients over 60 Years old. Ann Rehabil Med 2016;40:666-674. 27606273 10.5535/arm.2016.40.4.666 PMC5012978

[25] Kuru T Olçar HA: Effects of early mobilization and weight bearing on postoperative walking ability and pain in geriatric patients operated due to hip fracture: A retrospective analysis. Turk J Med Sci 2020;50:117-125. 31742370 10.3906/sag-1906-57

[26] Magaziner J Hawkes W Hebel JR : Recovery from hip fracture in eight areas of function. J Gerontol Ser A: Biol Sci Med Sci 2000;55:M498-M507. 10995047 10.1093/gerona/55.9.m498

[27] Fitzgerald M Blake C Askin D Quinlan J Coughlan T Cunningham C: Mobility one week after a hip fracture–can it be predicted?. Int J Orthop Trauma Nurs 2018;29:3-9. 29602677 10.1016/j.ijotn.2017.11.001

[28] Mariconda M Costa GG Cerbasi S : Factors predicting mobility and the change in activities of daily living after hip fracture: A 1-year prospective group study. J Orthop Trauma 2016;30:71-77. 26817573 10.1097/BOT.0000000000000448

[29] Klestil T Röder C Stotter C : Impact of timing of surgery in elderly hip fracture patients: A systematic review and meta-analysis. Sci Rep 2018;8:13933. 30224765 10.1038/s41598-018-32098-7 PMC6141544

[30] Nayar SK Marrache M Bressner JA Raad M Shafiq B Srikumaran U: Temporal trends in hip fractures: How has time-to-surgery changed?. Arch Bone Joint Surg 2021;9:224-229. 34026941 10.22038/abjs.2020.46195.2268 PMC8121027

[31] Caesar U Karlsson J Hansson E. Incidence and root causes of delays in emergency orthopaedic procedures: A single-centre experience of 36,017 consecutive cases over seven years. Patient Saf Surg. 2018;12, 2. 29344088 10.1186/s13037-018-0149-1 PMC5763611

[32] Cornwall R Gilbert MS Koval KJ Strauss E Siu AL: Functional outcomes and mortality vary among different types of hip fractures: A function of patient characteristics. Clin Orthop Relat Res 2004;425:64-71. 10.1097/01.blo.0000132406.37763.b3 PMC1455528 15292789

[33] Takahashi A Naruse H Kitade I : Functional outcomes after the treatment of hip fracture. PLoS One 2020;15:e0236652. 32730298 10.1371/journal.pone.0236652 PMC7392284

[34] Koval KJ Aharonoff GB Rokito AS Lyon T Zuckerman JD: Patients with femoral neck and intertrochanteric fractures. Are they the same?. Clin Orthop Relat Res 1996;330:166-172. 10.1097/00003086-199609000-00020 8804287

[35] Mautalen CA Vega EM Einhorn TA: Are the etiologies of cervical and trochanteric hip fractures different? Bone 1996;18:133S-137S. 8777078 10.1016/8756-3282(95)00490-4

[36] Sernbo I Johnell O: Background factors in patients with hip fractures--differences between cervical and trochanteric fractures. Compr Gerontol A 1987;1:109-111. 3453291

[37] Hayat Z Varacallo M: Surgical Management of Femoral Neck Fractures. Treasure Island (FL), StatPearls Publishing, 2022. 30855824

[38] Kristensen MT: Hip fracture-related pain strongly influences functional performance of patients with an intertrochanteric fracture upon discharge from the hospital. PM&R 2013;5:135-141 23182336 10.1016/j.pmrj.2012.10.006

[39] Lu Y Uppal HS: Hip fractures: Relevant anatomy, classification, and biomechanics of fracture and fixation. Geriatr Orthop Surg Rehabil 2019;10:215145931985913 10.1177/2151459319859139 PMC6610445 31321116

[40] Dolatowski FC Frihagen F Bartels S : Screw fixation versus hemiarthroplasty for nondisplaced femoral neck fractures in elderly patients: A multicenter randomized controlled trial. J Bone Joint Surg 2019;101:136-144. 30653043 10.2106/JBJS.18.00316

