# Supplementary material for: Ambulation Distance Within 72 Hours after Surgical Management Is a Predictor of 90-Day Ambulatory Capacity in Elderly Patients with Hip Fracture
Source: J Am Acad Orthop Surg Glob Res Rev. 2023 Aug 22;7(8):e23.00079. doi: 10.5435/JAAOSGlobal-D-23-00079 (PMC10445784; doi:10.5435/JAAOSGlobal-D-23-00079)
Supplement: Supplementary file 1 [file jagrr-7-e23.00079-s001.docx]

Supplemental Table. Patient Characteristics Stratified According to Ambulating 5 feet within 72 hours

|  | Time to Ambulate 5 feet | |
| --- | --- | --- |
|  | Early ambulatory (n = 84) | Minimally ambulatory  (n = 86) |
| Age  Mean (SD) | 78 (8.75) | 82.5 (9) |
| Sex | | |
| Male    Female | 30 (35.7%)  54 (64.3%) | 25 (29.1%)  61 (70.9%) |
| Charleston Comorbidity Index  Mean (SD) | 5.33 (2.28) | 5.59 (2.14) |
| Fracture Type | | |
| Femoral Neck    Pertrochanteric | 41 (48.8%)  43 (51.2%) | 30 (34.9%)  56 (65.1%) |
| Classifications (AO/OTA and Garden) |  |  |
| Garden  1  2  3  4  AO/OTA  Intertrochanteric  31-A1.1/2/3  31-A2.1/2/3  31-A3.1/2/3  Subtrochanteric  31-A1.1/2/3  31-A2.1/2/3  31-A3.1/2/3  31-B3.1/2/3  31-C1.1/2/3 | 21 (25.0%)  4 (4.8%)  8 (9.5%)  8 (9.5%)  19 (22.6%)  10 (11.9%)  6 (7.1%)  3 (3.6%)  2 (2.4%)  1 (1.2%)  0 (0%)  2 (2.4%) | 5 (5.8%)  5 (5.8%)  7 (8.1%)  13 (3.5%)  22 (25.6%)  13 (15.1%)  15 (17.4%)  3 (3.5%)  0 (0%)  1 (1.2%)  1 (1.2%)  1 (1.2%) |
| Surgery | | |
| Cephalomedullary Nail    Percutaneous Screw Fixation    Sliding Hip Screw    Arthroplasty | 36 (42.9%)  24 (28.6%)  10 (11.9%)  14 (16.7%) | 50 (58.1%)  5 (5.8%)  10 (11.6%)  21 (24.4%) |
| Pertrochanteric Fracture Fixation | | |
| Cephalomedullary Nail  Sliding Hip Screw | 36 (83.7%)  7 (16.3%) | 50 (89.3%)  6 (10.7%) |
| Femoral Neck Fracture Fixation | | |
| Percutaneous Screw Fixation  Sliding Hip Screw  Arthroplasty | 24 (58.5%)  3 (7.3%)  14 (34.1%) | 5 (16.7%)  4 (13.3%)  21 (70%) |
| Reoperation |  |  |
| Fixation Failure (malunion/nonunion/screw cutout)  Avascular Necrosis  Infection  Painful Hardware | 2 (2.4%)  3 (3.6%)  0 (0%)  1 (1.2%) | 3 (3.5%)  0 (0%)  1 (1.2%)  2 (2.3%) |
| Baseline Ambulatory Status | | |
| Ambulatory    Cane    Walker | 60 (71.4%)  7 (8.3%)  17 (20.2%) | 41 (47.7%)  16 (18.6%)  29 (33.7%) |
| Ambulatory Status 3 months after Surgery | | |
| Ambulatory    Cane    Walker    Non-ambulatory | 40 (48.2%)  12 (14.5%)  31 (37.3%)  0 (0%) | 7 (8.8%)  0 (0%)  70 (87.5%)  3 (3.8%) |
| Days from Injury to Surgery  Mean (SD) | 2.42 (4.78) | 1.81 (2.24) |
| Days from Presentation to Surgery  Mean (SD) | 1.15 (2.09) | 0.84 (1.02) |
| Days from Surgery to Last Follow-Up  Mean (SD) | 183.1 (169.8) | 206.8 (221.7) |
| Ambulatory Distance (ft) at First Postoperative Inpatient Physical Therapy Session  Mean (SD) | 26.1 (46.5) | 0.36 (0.88) |
| Ambulatory Distance (ft) at Discharge  Mean (SD) | 62.1 (65.3) | 0.71 (1.6) |
